# Pharmacy

**Published:** 1837-10

**Authors:** 


### PHARMACY.

### *Improved Method of Manufacturing the Ung. Hydrarg. fort.* By M. Boutigny, Apothecary at Evreux.

A pound of mercury, with two ounces of oil of turpentine, is put into a ten-ounce flask, which is then carefully corked; the bottle is well shaken for half an hour. The heterogeneous mixture is then poured into a marble mortar, furnished with a wooden pestle, and actively and incessantly triturated for twelve hours with one pound of lard: if globules are discoverable the next day, the trituration must be recommenced. Two days generally suffice. The advantages gained by this method are not unimportant: 1st, economy in time; 2d, the employment of fresh lard; 3d, the absence of foreign bodies in the composition; for the volatile oil is evaporated ere the proceeding is terminated, with the exception, at least, of a slight odour of turpentine.

*Bulletin gen. de Thérap. Jan.* 1837.

